# Trastuzumab and Gemcitabine in Pretreated HER2 Overexpressing Metastatic Breast Cancer Patients: Retrospective Analysis of Our Series

**DOI:** 10.1155/2012/198412

**Published:** 2012-03-26

**Authors:** Vincenzo Di Lauro, Elena Torrisi, Ettore Bidoli, Daniela Quitadamo, Sara Cecco, Andrea Veronesi

**Affiliations:** ^1^Division of Medical Oncology C, Centro di Riferimento Oncologico, 33081 Aviano, Italy; ^2^Epidemiology Unit, Centro di Riferimento Oncologico, 33081 Aviano, Italy; ^3^Clinical Trial Office, Centro di Riferimento Oncologico, 33081 Aviano, Italy; ^4^Pharmacy Unit, Centro di Riferimento Oncologico, 33081, Aviano, Italy

## Abstract

Trastuzumab-based regimes improved clinical outcome in women with overexpressing HER2 metastatic breast cancer, mainly due to the availability of different combination therapies, clinically active and well tolerated. In this study we retrospectively evaluated clinical activity and toxicity of trastuzuamb plus gemcitabine regimen in heavily pretreated HER2 positive metastatic breast cancer patients. Although the observed population was heavily pretreated, the evaluated regimen was notably effective in terms of response rate, time to progression and survival, with very mild toxicity. These data suggest that in over expressing HER2 metastatic breast cancer patients, sequential trastuzumab based chemotherapeutic regimens can achieve good response rate with prolonged TTP in responding patients, even after other target therapy such as lapatinib based combinations.

## 1. Introduction

Human epidermal growth factor receptor 2 (HER2) is a transmembrane tyrosine kinase which is crucial in cell growth and differentiation.

The HER2 gene is amplified in 15–20% of human breast cancers, and this feature is a well-known poor prognostic factor [1–3].

Trastuzumab (Herceptin Roche, Basel, Switzerland), a humanised monoclonal antibody with specificity for HER2, changed the natural history of HER2 overexpressing breast cancer, in the adjuvant setting as well as in metastatic disease.

After phase II studies, the results of important phase III randomized trial enabled the registration of regimen of weekly paclitaxel plus trastuzumab by Food and Drug Administration (FDA) for HER2-positive metastatic breast cancer (MBC) [4–7].

Although the combination of taxane with trastuzumab is considered the reference regimen for HER2 overexpressing MBC, other combination therapies were evaluated in extensive phase II trials [8].

In patients with progressive disease after first-line trastuzumab-based regimens, a current clinical practice is to continue trastuzumab changing the chemotherapeutic partner.

Therefore, the identification of different combinations of trastuzumab-based regimens for sequential use is important. 

In a previous study, we tested efficacy and tolerability of trastuzumab plus vinorelbine (VNR) regimen in first- and second-line setting for HER2 overexpressing MBC, with value for response rate (RR) of 78%, median time to progression (TTP) of 9 months, and median overall survival (OS) of 28 mounths. Very mild toxicity was observed [9].

Our results were in agreement with those previously published [10–19]. They were recently confirmed in two prospective and randomized trials, such as TRAVIOTA Study and HERNATA study, where the trastuzumab plus VNR regimen was not inferior then trastuzumab plus docetaxel/paclitaxel regimen in terms of anticancer activity and had less significant adverse events in first-line setting [20, 21].

Based on preclinical studies indicating synergistic activity originated by trastuzumab and gemcitabine (GEM) [22] and considering phase II study, where trastuzumab plus GEM regimen was effective and well tolerated [23], we chose this combination for pre treated patients in our clinical practice.

 In our study, the outcome of patients treated with trastuzumab/GEM regimen was retrospectively evaluated in order to asses the efficacy and the tolerability of this regimen.

This retrospective study was approved by the local Institution Review Board. The findings are the subject of the present report.

## 2. Patients and Methods

 We obtained, from our central pharmacy data base, the list of women with metastatic breast cancer HER2 3+ by IHC, or 2+ and positive for HER2 gene amplification, treated with GEM 1250 mg/m^2^on days 1 and 8 of a 21-day cycle plus trastuzumab 4 mg/kg loading dose and then 2 mg/kg weekly.

The main characteristics of treated patients are reported in Table 1. Sixty percent of patients had received three or more previous chemotherapy regimens for metastatic disease and 28% two-chemotherapy regimens, including trastuzumab-based chemotherapy (90%) and lapatinib plus capecitabine (41%).

Sixty-nine percent of patients had more than two metastatic sites, one visceral site was reported for 7% of population, bone only for 7% and nodal and/or soft tissue for 17% of patients.

Metastasis to Central Nervous System (CNS) was present in 14% of patient's population, under clinical control after radiation therapy.

We used a 21-day cycle, with trastuzumab given weekly at a loading dose of 4 mg/kg intravenously (IV) over 90 minutes, and at 2 mg/kg IV in 30 minutes thereafter.

To avoid infusion syndrome, the first trastuzumab infusion was premedicated with chlorphenamine 100 mg IV and patients were monitored for 60 minutes after infusion.

GEM was given at the dose of 1250 mg/m^2^ on days 1 and 8 of 21-day cycle on the same day, after the infusion of trastuzumab.

Complete blood counts with differential were obtained weekly and liver and renal tests were performed every 3 weeks.

Tumour marker, Ca 15-3, was performed periodically if it was elevated at the beginning of the treatment.

The dose of GEM was reduced by 50% if neutrophil absolute count ranged between 1,000 and 1,500/mL. and it was omitted in case of lower neutrophil values.

No trastuzumab dose modification was foreseen for low neutrophil absolute counts.

No other concomitant antineoplastic treatment was delivered, except bisphosphonates for women with bone metastases.

According to our clinical practice, tumour response was assessed every 3 cycles, performing the clinical and radiological examinations.

The therapy lasted until unacceptable toxicity or progression.

Response and toxicity were assessed according to WHO standard and NCI criteria [24, 25].

By retrospective analysis, primary end points were RR and TTP, calculated from the first day of therapy until progression or relapse.

Secondary end points were median duration of response, estimated from date of documented complete response (CR) or partial response (PR) until relapse or progression, and OS, calculated from the beginning of therapy until death.

## 3. Results

A median number of 7 cycles of trastuzumab/GEM regimen were delivered (range 2–17). Forty-one patients were assessed for response and toxicity.  Table 2 shows tumour response. We observed 1 CR (3%) and 18 PRs (44%) for an overall 47% RR. Stable disease (SD) longer than 6 months was observed in 7 patients (17%), for an overall clinical benefit (CR+PR+SD) of 64%. Fifteen patients progressed (36%). 

**Figure 1:**
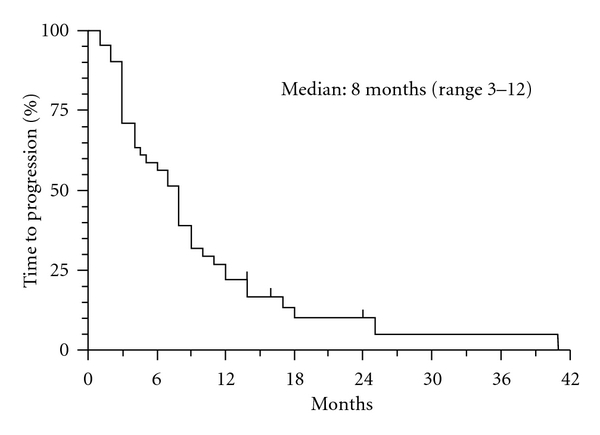
TTP (Time to progression).

**Figure 2:**
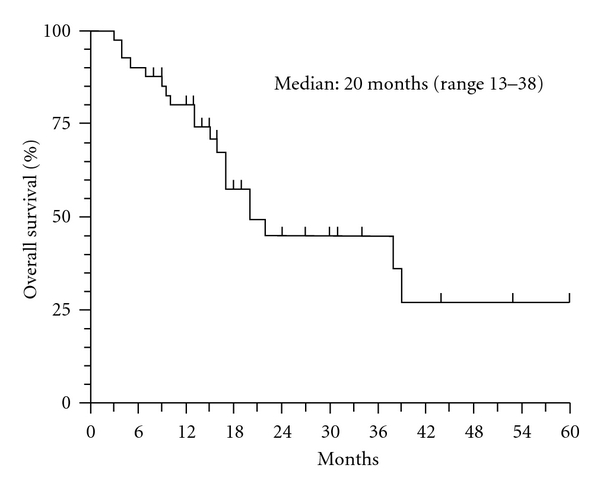
OS (overall survival).

No difference was noted about visceral and not visceral disease. Median duration of PR was 6 months (95% CI, 1,5–18 months) and one CR lasted 10 months. Median TTP and OS are reported in Figures 1 and 2.

In the whole population median TTP was 8 months (95% CI, 3–12) and median OS was 20 months (95% CI, 13–38). The toxicity of 21 day regimen was very mild. No toxicity of grade 4 was detected. Neutrophil absolute counts were less than 1000/*μ*L only for seven patients (17%) and omission of scheduled GEM dose was applied.

 In these cases, we did not observe neutropenic fever and spontaneous recovery occurred promptly, without supportive measures or clinical complications.

For five patients (12%) the dose of GEM was reduced to 80% during the duration of therapy, due to frequent reductions and omissions of the planned dose of GEM.

Nonhaematological toxicity was mild and no G3 or 4 toxicities were reported.

Gastrointestinal side effects, such as nausea/vomiting or stomatitis were mild and no alopecia was reported, which increased compliance to treatment.

## 4. Discussion

Trastuzumab-based regimen improved clinical outcome of women with HER2 overexpressing MBC, mainly due to the availability of different combination regimens, clinically active, and well tolerated.

Phases II and III trials have demonstrated the activity and safety of trastuzumab administered with chemotherapy, including weekly taxane, as well as VNR, platinum salts, GEM, and a variety of other combinations.

Today, the optimal trastuzumab-based regimen and the appropriate sequencing of these combination therapies are not know.

The choice of the regimen to treat the HER2-positive patients has to consider previous chemotherapy regimens and the risk benefit ratio of these therapies [26].

Although the combination of taxane with trastuzumab is considered the reference therapy for HER2 overexpressing MBC, the recent published data of Traviota and Hernata study support the use of trastuzumab plus weekly VNR, as appropriate first-line regimen, as well as second-line regimen [9–21].

In a recent study, Yardley investigated the activity and toxicity of combination therapy with trastuzumab and GEM as first- and second-line treatment for patients with HER2 overexpressing MBC and this regimen appears less active than trastuzumab in combination with either taxanes or VNR, with RR of 30% and TTP of 4 months [27].

In phase II trial, O'Shaughnessy described a RR of 44%, with TTP and OS of 5,8 and 14,7 months, respectively for combination of trastuzumab and GEM in pre-treated HER2 positive MBC, with very mild toxicity [23]. 

Even with the limitations arising from retrospective character of our analysis, our results agree to those which reported RR of 47%, TTP of 8 months and OS of 20 months and confirm that the regimen evaluated is effective and well tolerated, even in this heavily pretreated population.

Notably, all patients that showed PRs were pretreated by trastuzumab-based regimens mostly trastuzumab plus weekly paclitaxel or trastuzumab plus weekly VNR, or both.

Among 17 pretreated patients also by lapatinib plus capecitabine, we described 11 PRs (65%) and 4 SD (23%) with a clinical benefit of 88%, while two patients progressed (12%).

The unique CR was reported in a patient pretreated in first line by docetaxel 21 day regimen and by weekly trastuzumab and VNR as second line chemotherapy.

This CR occurred in patient with locoregional disease and lasted 10 months.

To continue trastuzumab-based regimen after disease progression is still debated, due to a substantial lack of scientific evidence, since randomized studies adequately powered have not been completed.

Recently, von Minckwitz et al. reported results of the first randomized trial, assigning patients who experience progression while receiving a trastuzumab-containing regimen, to stop or continue this antibody with the institution of capecitabine therapy [28].

They enrolled 78 patients per arm, and although this number is much less than the 482 intended; they showed a better TTP in favour of combination (5.6 versus 8.2 months; **P** = .0338), and better RR with doublet therapy (27% versus 48%; **P** = .0115), but OS was not statistically significant.

The small number of patients in this trial was due to slowly accrual and early closing, because of availability of lapatinib plus capecitabine data [29], and it limited the statistical power of these observations.

Although definitive data in this setting are lacking, it is becoming clear that to continue HER2 blockade is very important in patients with HER2 overexpressing MBC, and clinicians often reinstitute trastuzumab combined with other cytotoxic drugs after progression on lapatinib based therapy.

With increasing evidence of activity of newer compounds, such as trastuzumab-DM 1 [30], pertuzumab [31], and neratinib [32], we will have to figure out the best sequence of these therapies, identify mechanisms of resistance, and then find a way to individualize therapy.

## Figures and Tables

**Table 1:** Patient's characteristics.

	*N*	%
No. of patients	41	100
Median age	54 (33–75)	

PS (ECOG)		
0	30	73
1	11	27

Histology		
Ductal	36	88
Lobular	5	12

Hormonal Receptor status		
EgR+PgR+	24	58
EgR+PgR−	4	10
EgR−PgR+	1	2
EgR−PgR−	12	29

HER2 Status		
3**+ **	32	78
2**+ **CISH+	9	22

Stage at diagnosis		
I	3	7
IIA	8	20
IIB	2	5
IIIA	16	39
IIIB	5	12
IIIC	5	12
IV	2	5

Metastatic site		
**N** **o** **d** **a** **l** **i** **n** **v** **o** **l** **v** **e** **m** **e** **n** **t** ± **s** **o** **f** **t** **t** **i** **s** **s** **u** **e**	7	17
As bone only	3	7
1 visceral site (liver, lung, and pleura) only	3	7
Metastatic sites ≥2	28	69
Metastasis to CNS	6	14
Prior adjuvant CT	38	92
Adjuvant trastuzumab	3	7
Prior CT for metastatic disease	41	100
1 regimen	5	12
2 regimens	11	28
**≥**3 regimens	25	60
**P** **r** **i** **o** **r** **t** **r** **a** **s** **t** **u** **z** **u** **m** **a** **b** − **b** **a** **s** **e** **d** **C** **T**	37	90
**P** **r** **i** **o** **r** **l** **a** **p** **a** **t** **i** **n** **i** **b** + **c** **a** **p** **e** **c** **i** **t** **a** **b** **i** **n** **e**	17	41

ECOG: Eastern Cooperative Oncology Group, EgR: Estrogen Receptor, PgR: Progesteron Receptor, CISH: Chromogenic In Situ Hybridisation, CT: Chemotherapy, CNS: Central Nervous System.

**Table 2:** Tumor Response (*n* = 41).

	*N*	%
Overall response	19	47
Complete response	1	3
Partial response	18	44
Stable disease ≥6 months	7	17
Clinical benefit	26	64
Progression	15	36
